# A novel taxon selection method, aimed at minimizing recombination, clarifies the discovery of a new sub‐population of *Helicobacter pylori* from Australia

**DOI:** 10.1111/eva.12864

**Published:** 2019-09-18

**Authors:** Binit Lamichhane, Michael J. Wise, Eng Guan Chua, Barry J. Marshall, Chin Yen Tay

**Affiliations:** ^1^ Helicobacter pylori Research Laboratory Marshall Centre for Infectious Disease Research and Training School of Biomedical Sciences University of Western Australia Perth WA Australia; ^2^ Department of Computer Science and Software Engineering University of Western Australia Perth WA Australia

**Keywords:** core genomes, *Helicobacter pylori*, phylogeography, recombination, taxon selection

## Abstract

We present a novel method for taxon selection, the aim being to minimize problems arising from highly recombinant species such as *Helicobacter pylori*. *Helicobacter pylori* has accompanied modern‐human migration out of Africa and is marked by a phylogeographic strain distribution, which has been exploited to add an extra layer of information about human migrations to that obtained from human sources. However, *H. pylori's* genome has high sequence heterogeneity combined with a very high rate of recombination, causing major allelic diversification across strains. On the other hand, recombination events that have become preserved in sub‐populations are a useful source of phylogenetic information. This creates a potential problem in selecting representative strains for particular genetic or phylogeographic clusters and generally ameliorating the impact on analyses of extensive low‐level recombination. To address this issue, we perform multiple population structure‐based analyses on core genomes to select exemplar strains, called ‘quintessents’, which exhibit limited recombination. In essence, quintessent strains are representative of their specific phylogenetic clades and can be used to refine the current MLST concatenation‐based population structure classification system. The use of quintessents reduces the noise due to local recombination events, while preserving recombination events that have become fixed in sub‐populations. We illustrate the method with an analysis of core genome concatenations from 185 *H. pylori* strains, which reveals a recent speciation event resulting from the recombination of strains from phylogeographic clade hpSahul, carried by Aboriginal Australians, and hpEurope, carried by some of the people who arrived in Australia over the past 200 years. The signal is much clearer when based on quintessent strains, but absent from the analysis based on MLST concatenations.

## 1 INTRODUCTION

### 1.1 *Helicobacter pylori* and its highly recombinant genome

*Helicobacter pylori* is a major gastric pathogen that infects about half of the human population in the world. The infection rate can be as high as 90% in developing countries, though it is usually less than 30% in developed countries (Covacci, Telford, Giudice, Parsonnet, & Rappuoli, 1999; Linz et al., 2007). The general prevalence of *H. pylori* in Australia has been reported as ranging from 15% to 30% (Lin, Lambert, Nicholson, Lukito, & Wahlqvist, 1998; Robertson, Cade, Savoia, & Clancy, 2003). However, the prevalence among Aboriginal Australians can reach as high as 76% (Windsor et al., 2005). *Helicobacter pylori* is believed to be transmitted between close family members via oral–oral, gastric–oral or faecal–oral routes, usually during childhood and usually from mother to children (Goh, Chan, Shiota, & Yamaoka, 2011). It has been coevolving with its human host for more than 120,000 years and migrated with us, which has led to the emergence of different phylogeographic genotypes over time. Therefore, *H. pylori* has also become a useful genetic marker, providing information about relationships between human ethnic populations and the human migration history (Linz et al., 2007; Tay et al., 2009). However, the sequence heterogeneity within *H. pylori* is very high (Tay et al., 2009), likely due to the lack of a proof‐reading function in DNA polymerase I (Garcia‐Ortiz et al., 2011) together with a very high recombination rate that facilitates the exchange of genes between genetically different isolates (Baltrus, Guillemin, & Phillips, 2007; Didelot et al., 2013).

### 1.2 Studying *H. pylori* evolution and population structure

Multilocus sequence typing (MLST), based on concatenated fragments of 7 housekeeping genes, has long been used to reveal the evolutionary history of *H. pylori* and its correlation with the human Out‐of‐Africa migration hypothesis (Linz et al., 2007; Maixner et al., 2016; Moodley et al., 2009). On top of this, the program STRUCTURE, using Bayesian methods, has been widely used to deduce population structure based on MLST data. In particular, STRUCTURE has been used in many studies to determine the population structure of many human pathogens, including *H. pylori* (Falush et al., 2003; Maixner et al., 2016; Tay et al., 2009). To date, *H. pylori* has been classified into 7 distinct populations that are associated with particular geographic areas: hpAfrica2, hpAfrica1, hpNEAfrica, hpEurope, hpAsia2, hpEastAsia and hpSahul (Falush et al., 2003; Linz et al., 2007; Moodley et al., 2009). hpSahul is named after the ancient Sahul continent, that is mainland Australia, Tasmania and New Guinea, which were joined from 100 kya (100,000 years ago) to relatively recent times, some 31,000–37,000 years ago. hpSahul is only carried by Aboriginal Australians and is thought to have been split from the East Asia *H. pylori* population (Moodley et al., 2009) when Aboriginal Australians first migrated to Australia 65,000 years ago (Clarkson et al., 2017).

### 1.3 Genomic and taxon selection issues when studying *H. pylori* and other species with highly recombinant genomes

In studying *H. pylori*, we became aware that previous studies of the population structure of *H. pylori* have faced unresolved methodological issues. Most studies based on *H. pylori* have, thus far, been based on MLST concatenations (Linz et al., 2007; Maixner et al., 2016; Moodley et al., 2009; Tay et al., 2009), though with the development of cost‐effective, high‐throughput whole‐genome sequencing technology, it is possible to undertake phylogenetic studies using concatenations of core genes (Gressmann et al., 2005) or whole genomes (Kumar et al., 2015). However, even this may not give clear‐cut information due to the highly recombinant nature of *H. pylori*. In particular, recombination events can be problematic for phylogenetic analyses as they break the assumption of clonal descent (Didelot & Falush, 2007). On the other hand, issues arising from the fact that different genes face different evolutionary pressures (Wise, 2013) can affect single‐gene studies or studies based on concatenations of just a few genes.

In the phylogeographic literature based on *H. pylori*, there have been, in essence, two approaches to dealing with species whose genomes evidence a high level of recombination. The first approach has been to tacitly ignore the problem. This approach is evident in the numerous MLST gene fragment‐based studies, but also in the whole‐genome studies, which are discussed above. The other approach is to use applications such as ClonalFrameML (Didelot & Wilson, 2015) or Gubbins (Croucher et al., 2015) which are given, or compute, a phylogenetic tree and then remove parts of the input sequence multiple alignments which are not consonant with an assumption of clonal descent. Currently, both of these programs only apply to nucleotide sequence data.

For this study, we sequenced 177 stains and, together with 8 well‐studied strains from the literature, then identified the core genomes of the total set of 185 *H. pylori* strains. Core genomes provide us with much better resolution and allow us to delimit strain diversity more precisely rather than have been possible with MLST‐based studies. However, core genomes can still exhibit considerable levels of recombination. To deal with this, using the core genomes, we identified exemplar strains from each *H. pylori* sub‐population that has limited, or no, recombination with the other sub‐populations. We have called these exemplars quintessents (to connote a set of primary objects, from which secondary objects are obtained by combination). In other words, in this study we have taken a different approach than ClonalFrameML and Gubbins, viewing the problem of limiting recombination as a taxon selection issue. There has been a considerable history of debate about how best to select taxa for phylogenetic analyses (see the review Nabhan and Sarkar (2012), for example). What is proposed here is that freedom from recombination could be one criterion.

## 2 MATERIALS AND METHODS

### 2.1 Selecting *H. pylori* strains and obtaining DNA

One hundred and fifty‐five *H. pylori* strains were isolated from patients attending Sir Charles Gairdner Hospital (Perth, Western Australia) for treatment of antibiotic‐resistant *H. pylori* infection. All were informed about the nature of study, and written consent was obtained from those who wished to proceed. The protocols were approved by the hospital's Human Research Ethics Committee. A further 22 strains, classed as hpSahul by MLST, originally obtained during the Windsor et al. (2005) study, with MLST sequences reported in Moodley et al. (2009), were fully sequenced in this study. Of these, strain HPJ023, sequenced in this study, was also studied by Montano et al., who named it ausabrJ05 (Montano et al., 2015).

The 177 strains were grown on 5% horse blood agar (HBA) plates as previously described (Lu et al., 2014). Genomic DNA was extracted from each *H. pylori* strain using DNeasy Blood and Tissue Kit (Qiagen) according to the manufacturer's instruction. The quality of the DNA samples was checked using NanoDrop 2000 Spectrophotometer and Qubit Fluorometer. The samples were then stored at −20°C until they were analysed.

### 2.2 DNA sequencing, genome assembly and determination of the core genome

1 ng of bacterial genomic DNA was used for genomic library preparation using Nextera XT protocol (Ver. September 2014). The libraries were then subjected to 250 bp paired‐end sequencing on a MiSeq Sequencer running version 1.1.1 MiSeq Control Software (Illumina Inc.). The de novo assembly of raw reads was performed using St. Petersburg genome assembler (SPAdes, Ver.3.8.2) (Bankevich et al., 2012). Contigs with coverage less than 10 times and length less than 500 were filtered. In addition to the above 177 *H. pylori* strains, eight well‐studied, publicly available complete genome sequences were also included in the analysis. FASTA format files of the strains 26695, G27, Sahul64, India7, Pecan4, SouthAfrica7, J99 and F57 sequences were obtained from NCBI via the genome browser (http://www.ncbi.nlm.nih.gov/genome/browse/). To ensure uniformity of approach, all 185 genomes, including the eight reference sequences, were annotated using Prokka (Ver. 1.11) (Seemann, 2014). Details of the respective genomes for the strains sequenced for this project can be found in Table S1; corresponding details for the strains from NCBI can be found in Table S2. The core genome of all 185 *H. pylori* strains was determined at the protein level using a best reciprocal BLAST heuristic implemented in the program Proteinortho v.5.1 (settings: ‐e = 1e−05, ‐p = blastp, ‐id = 50, ‐cov = 80, ‐conn = 0.1, ‐sim = 0.95) (Lechner et al., 2011). Genes present in 100% of the strains constituted the core genome, which consisted of 898 genes. Gene‐by‐gene alignments of single‐copy orthologous core genes were performed using MAFFT version 7.271 (Katoh & Standley, 2013) with options –maxiterate = 1,000 and ‐localpair. The SNPs were extracted using SNP sites (Page et al., 2016).

### 2.3 Determining population structures and the set of quintessents

The SNPs were subjected to STRUCTURE v.2.3.4 (Pritchard, Stephens, & Donnelly, 2000) analysis, which implements a Bayesian approach to deducing the population structure, based on an a priori fixed number of sub‐populations. The Markov chain Monte Carlo (MCMC) simulation underpinning STRUCTURE was run for 100,000 iterations, following a burn‐in of 100,000 iterations. A no‐admixture population model was used, supported by a correlated frequency model for allele frequencies (Porras‐Hurtado et al., 2013). To determine the number of sub‐populations, K, STRUCTURE was run for K ranging from 4 to 12, and each run was repeated 12 times. Structure Harvester v0.6.94 (Earl & vonHoldt, 2012) was then used to determine the optimal value of K, which occurred for K = 11. For the K = 11 data set, a strain was considered to be quintessent, that is an exemplar of a particular sub‐population, if it was assigned to that sub‐population with probability of at least 0.75 in at least 60% of the runs. We tried a number of combinations of the probability and run‐percentage cut‐offs, and selected this particular combination because it maximized the number and size of clades while also minimizing recombination. With the combination of 0.75 probability in at least 60% of the runs, 93 strains were identified as being quintessents. The set of quintessent strains is noted with a ‘*’ in Tables S1 and S2.

### 2.4 Phylogenetic and recombination analysis

Four different sequence data sets were created for this study: nucleotide concatenations of the 898 core genes for each of the 185 strains (the set labelled all nt), protein concatenations of the corresponding 898 core proteins for each of the 185 strains (all aa), MLST concatenations for each of the 185 strains (mlst) and protein concatenations from 472 minimally recombinant genes from 93 quintessent strains (quint aa). The 472 minimally recombinant genes were those whose Phi statistics were greater than or equal to 0.1. (The Pairwise Homoplasy Index, Phi, is discussed below, together with the interpretation of the *p*‐value threshold.) The MPI‐based, genome‐scale phylogenetic tree building application ExaML (Kozlov, Aberer, & Stamatakis, 2015) was used to create the trees for the different sequence data sets based on multiple sequence alignments created using Clustal Omega (Sievers et al., 2011). In each case, 500 bootstrapped trees were computed, with a gamma model for mutation rate heterogeneity across sites. A starting neighbour‐joining tree was created using Molecular Evolutionary Genetics Analysis software version 7.0 (MEGA 7.0) (Kumar, Stecher, & Tamura, 2016) with default parameter settings. The best of the trees computed by ExaML was then annotated with bipartition data—effectively bootstrap percentages—using RAxML (Stamatakis, 2014), and the final trees were visualized using Figtree (v1.4.3) (https://github.com/rambaut/figtree/releases).

The extent of recombination was measured using the Pairwise Homoplasy Index, Phi (Bruen, Philippe, & Bryant, 2006). Phi is a *p*‐value, related to the probability of rejecting the null hypothesis that there is no recombination in the set of aligned sequences being tested in a sliding window. In our experiments, the Phi statistic was based on a window size of 20 for amino acids or 60 for nucleotides, with a permutation test used to compute statistical significance. The window size was reduced from 100 to prevent the metric becoming saturated, and every gene in this very recombinogenic organism thus appearing to be recombinant.

Finally, to assess the phylogenetic tree distance between the new hpEuropeSahul clade (see below) and the nearest hpEurope or hpSahul taxon for each of the core genes, PhyML (Guindon & Gascuel, 2003) was used to create an unbootstrapped tree, but with topology and branch‐length optimization. A stand‐alone Python program was then used to compute the shortest distance from each hpEuropeSahul taxon to the nearest hpEurope taxon, and also to the nearest hpSahul taxon. The code for the program can be found at https://github.com/mw263/clade to clade distance.

### 2.5 Comparison of quintessents with ClonalFrameML

Comparison of quintessent selection with existing methods, for example ClonalFrameML, is complicated by the fact the methods are very different: ClonalFrameML only works with nucleotide sequences (genomes or gene concatenations), while optimal results for the quintessent method are obtained with amino acid data, though the method also works for nucleotide data. ClonalFrameML keeps the complete set of input sequences, but the length of the sequences has been reduced, while the quintessent method returns a reduced set of sequences, but with the sequence lengths unchanged. Therefore, in order to compare like with like, the data set all nt (see above) was input into ClonalFrameML to create the data set all nt cf, while the 93 strains, identified as quintessent, were taken from all nt to represent the quintessent data set. This data set was called quint nt. In other words, to the original suite of data sets described above: all nt, all aa, mlst and quint aa, were added all nt cf and quint nt. The starting tree created using MEGA 7.0 (described earlier) was the second input to ClonalFrameML.

As first comparison, cladistic information content, in the form of the dCITE metric (Wise, 2016), was computed for the two new data sets, all nt cf and quint nt, and compared with the correspondding data from the all nt data set. A second method for examining the two approaches was to compare the trees computed using ExaML (outlined above) for the six data sets—all against all—but focusing on all nt cf. The application TreeCmp (Bogdanowicz, Giaro, & Wróbel, 2012) was used to compare pairs of trees based on four metrics: Robinson–Foulds distance, Estabrook's quartet distance, Steel and Penny's path difference distance and the TreeCmp authors' own metric, matching split distance.

## 3 RESULTS AND DISCUSSION

### 3.1 General features and pan‐genome of *H. pylori* genomes

We sequenced 177 *H. pylori* genomes isolated from Australia, which represented all 7 known MLST‐based STRUCTURE clades. Eight complete genomes obtained from NCBI belonging to different MLST STRUCTURE clades were also included in the analysis. The 177 new genomes were sequenced with at least 100× coverage for each strain and assembled into between 19 and 86 contigs. In our study, *H. pylori* genome size ranged between 1.53 and 1.74 Mb. The genomes had an average G + C content of 38% and were predicted to encode between 1,443 and 1,658 genes. Summaries of genomes sequenced in this study are presented in Table S1. Orthologous group analysis resulted in 906 genes found to be present in all 185 strains and constituted the core genome. Among them, 898 were single‐copy orthologous genes with the concatenated length of 848 kbp. The number of accessory genes ranged from 495 to 710. The number of core genes found in this study is much lower than the 1,111 core genes found by Gressmann et al. (2005), 1,223 found by Fischer et al. (2010), the 1,226 genes found by Kumar et al. (2015), and 1,187 found by Kumar, Albert, Abkal, Siddique, and Ahmed (2017). Given that *H. pylori* is a panmictic species, the reduced core genome suggests that our samples represented a greater breadth of *H. pylori* strains. However, the fact that these are draft genomes may also have had an impact on size of the core genome.

### 3.2 Discrepancy in phylogenetic analysis based on MLST versus core genomes

**Figure 1:**
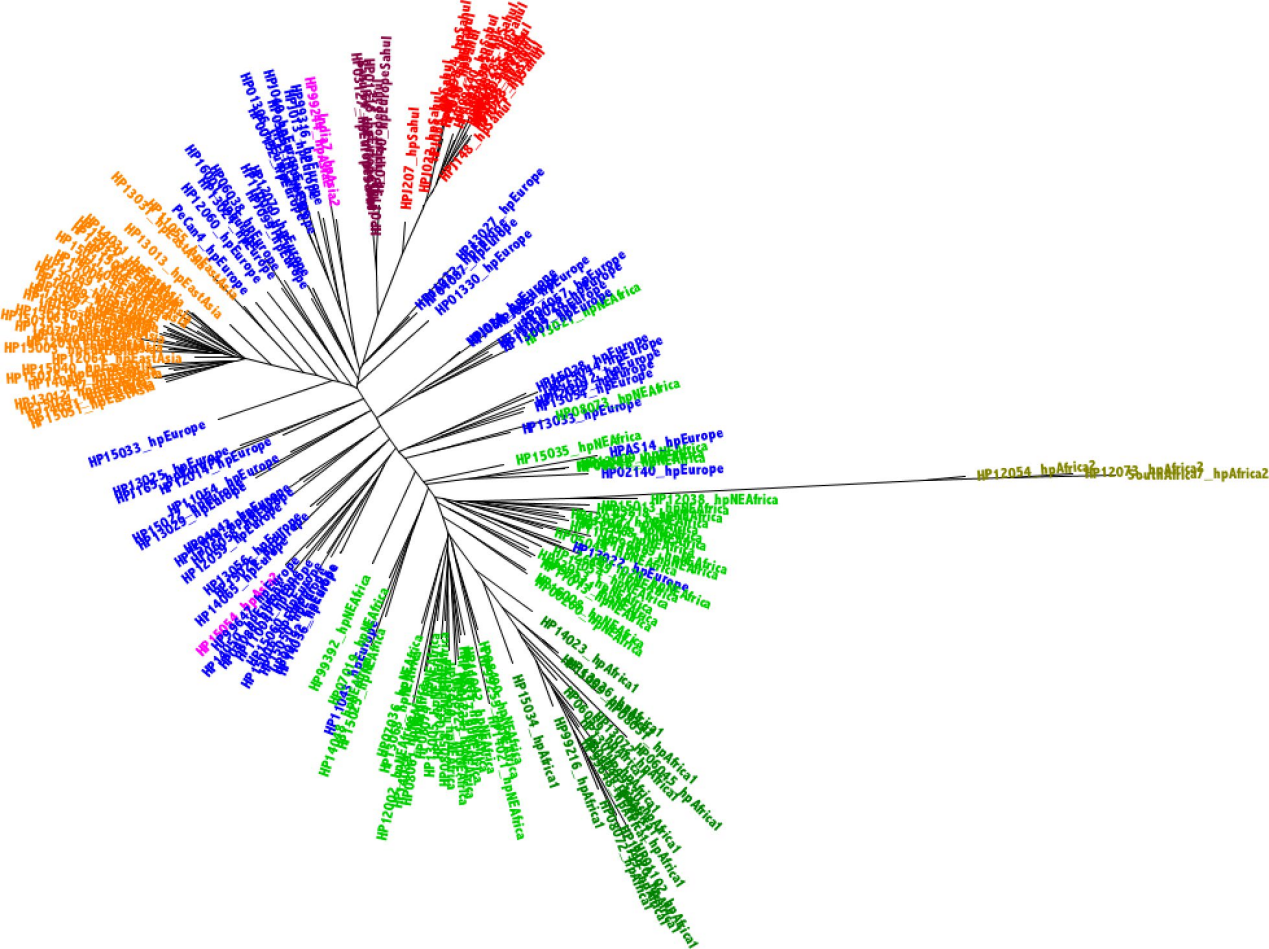
185 Strains MLST tree. A phylogenetic tree was created by ExaML, based on MLST gene fragment concatenation data from all 185 strains. A starting tree was created using Mega. Five hundred bootstrapped trees were computed, with the best (i.e., lowest absolute value log likelihood) shown here. The taxa have been labelled, both in the labels and by colour, according to the highest percentage STRUCTURE group, based on the best (lowest absolute value log likelihood) of 12 runs for k = 7 bins (based on suggestion from Moodley et al. (2009)). The clade colours are as follows: hpAfrica2 (olive), hpNEAfrica (bright green), hpAfrica1 (dark green), hpEurope (blue), hpAsia2 (pink), hpEastAsia (orange) and hpSahul (red). The sub‐clade of hpSahul that we now discover is a new clade, hpEuropeSahul, is shown in maroon

**Figure 2:**
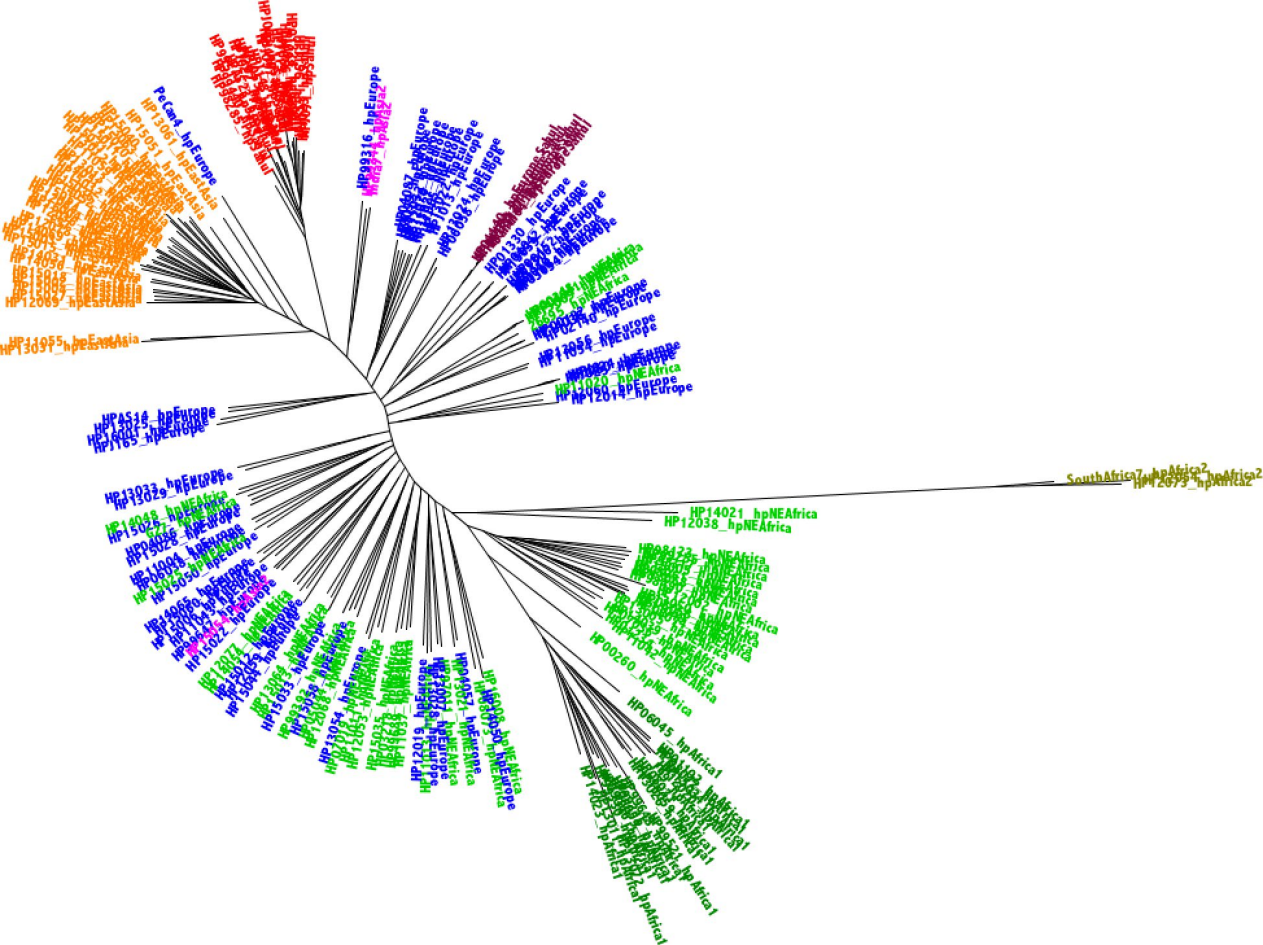
185 Strains core genome tree. A phylogenetic tree was created by ExaML, based on core genome concatenations of 898 genes from all 185 strains. A starting tree was created using Mega. Five hundred bootstrapped trees were computed, with the best (i.e., lowest absolute value log likelihood) shown here. The taxa have been labelled, both in the labels and by colour, according to the highest percentage STRUCTURE group, based on the best (lowest absolute value log likelihood) of 12 runs for k = 7 bins (based on suggestion from Moodley et al. (2009)). The clade colours are as follows: hpAfrica2 (olive), hpNEAfrica (bright green), hpAfrica1 (dark green), hpEurope (blue), hpAsia2 (pink), hpEastAsia (orange) and hpSahul (red). The sub‐clade of hpSahul that we now discover is a new clade, hpEuropeSahul, is shown in maroon

**Figure 3:**
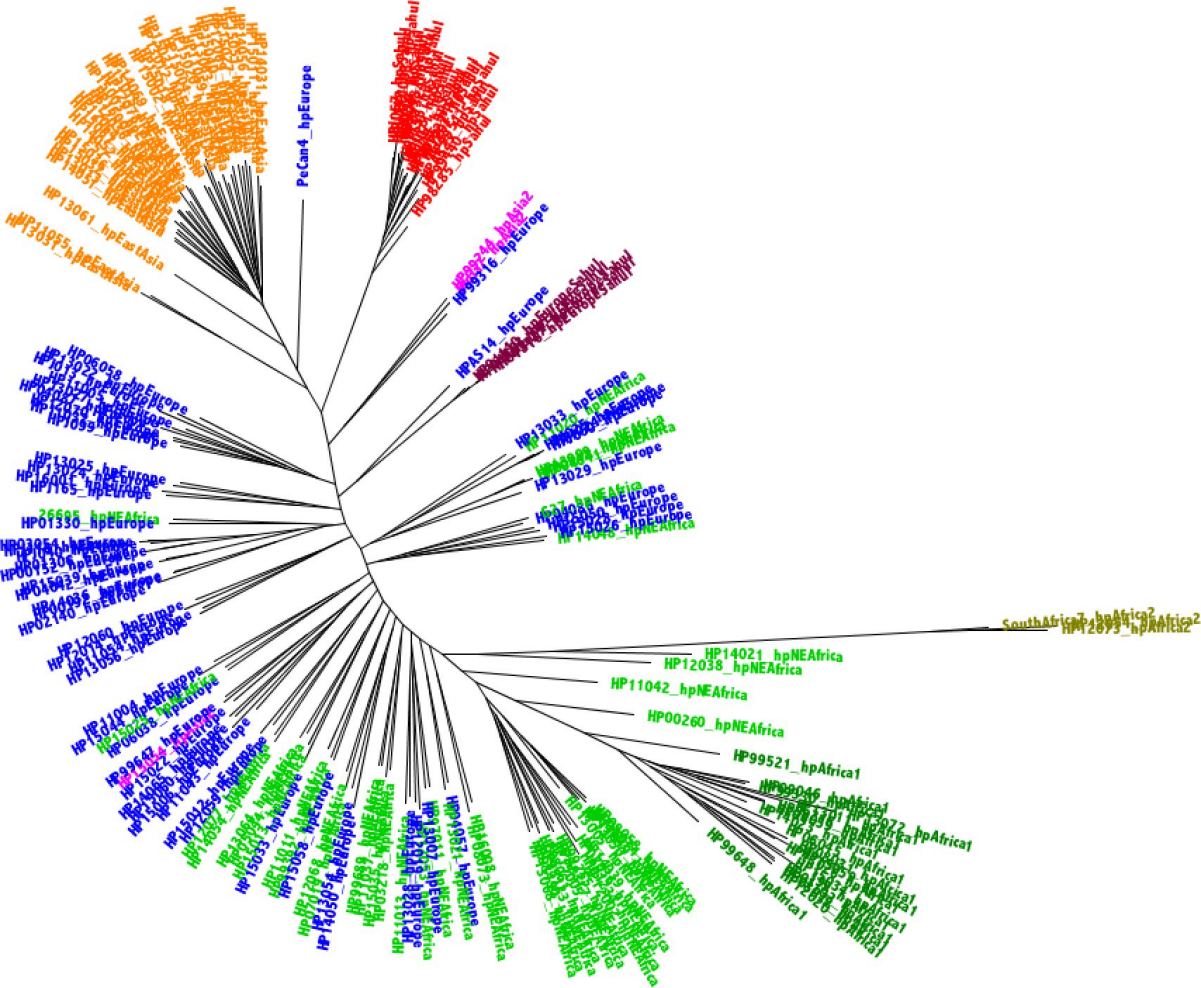
185 Strains core proteome tree. A phylogenetic tree was created by ExaML, based on core proteome concatenations of 898 protein sequences from all 185 strains. A starting tree was created using Mega. Five hundred bootstrapped trees were computed, with the best (i.e., lowest absolute value log likelihood) shown here. The taxa have been labelled, both in the labels and by colour, according to the highest percentage STRUCTURE group, based on the best (lowest absolute value log likelihood) of 12 runs for k = 7 bins (based on suggestion from Moodley et al. (2009)). The clade colours are as follows: hpAfrica2 (olive), hpNEAfrica (bright green), hpAfrica1 (dark green), hpEurope (blue), hpAsia2 (pink), hpEastAsia (orange) and hpSahul (red). The sub‐clade of hpSahul that we now discover is a new clade, hpEuropeSahul, is shown in maroon

The phylogenetic tree created from the concatenated MLST genes demonstrated a clustering pattern similar to those reported by many previous studies (Achtman et al., 1999; Falush et al., 2003; Linz et al., 2007; Moodley et al., 2009). Briefly, strains were clustered into 7 major *H. pylori* population types (Figure 1, in which each strain is coloured by the highest percentage STRUCTURE group). By contrast, the phylogenetic analysis using concatenated 898 core genes revealed clustering that is only partially consistent with that found using MLST; among other differences, five hpSahul strains namely, HP01140, HP01316, HP03127, HP01193 and Sahul64, were grouped together with the hpEurope strains (Figure 2, based on nucleotide data, and Figure 3, based on the corresponding protein sequence concatenations), compared with the MLST‐based tree (Figure 1), where these strains shared an ancestral node with hpSahul. In particular, based on MLST assignment, Sahul64 has previously been used as a reference genome to represent the hpSahul *H. pylori* population (Lu et al., 2014). However, in this study, it was among the five strains that were grouped with hpEurope. Interestingly, all of these five strains were isolated from unrelated Aboriginal Australians, suggesting these strains are the result of recombination between hpSahul and hpEurope *H. pylori* populations that are now being stably inherited. Discrepancies between the phylogenetic trees derived from MLST gene fragment concatenations and core genome concatenations have also been reported in previous studies (Gressmann et al., 2005; Munoz‐Ramirez et al., 2017), but this is the first time it has been reported to the extent where strains are grouped in a totally unrelated cluster.

Whole‐genome sequencing has broadened the opportunity to include more genetic information, which increases the resolution in population‐based phylogenetic studies (Qin et al., 2016). Therefore, using a broader spectrum of genes should be preferred, rather than MLST concatenations of gene fragments, in order to increase the cladistic information (Wise, 2016) and thereby improve the resolution in phylogenetic analyses. However, use of whole‐genome data is problematic in highly recombinant species such as *H. pylori*.

### 3.3 Selection and testing of quintessents

The presence of a remarkable degree of genetic variability in *H. pylori* is driven by frequent recombination and a high mutation rate (Falush et al., 2001; Kersulyte, Chalkauskas, & Berg, 1999; Suerbaum & Josenhans, 2007; Suerbaum et al., 1998). As a result, *H. pylori* largely lacks a clonal structure—except due to founder effects—and has been characterized as ‘weakly clonal’ (Achtman et al., 1999). On the other hand, the high recombination rate and ability of *H. pylori* to undergo frequent mutation lead to only partial linkage disequilibrium between polymorphic loci, which can provide additional information for population genetic analysis (Didelot et al., 2013; Suerbaum & Josenhans, 2007). However, this may cause substantial problems in the selection of strains representing particular sub‐populations for comparative genomic and phylogeographic studies.

In this study, to determine the set of strains that are exemplars of a particular sub‐population—which we have called quintessents—we ran the program STRUCTURE on the 270,782 SNPs extracted from the core genomes using an admixture model, where the individuals have inherited some fraction of their genomes from ancestors in up to K = 11 sub‐populations. Ninety‐three strains were found to belong to a particular structure cluster with probability of at least 0.75 in 60% of the runs and were therefore considered to be quintessents. We believe it is necessary to distinguish between ancient recombinations that have been preserved in particular sub‐populations and local recent recombination noise. In this context, the significance of quintessents is that, with limited recombination evident, we presume the quintessents to be closer to the founder strains for their respective sub‐populations. Viewed another way, quintessents are sub‐populations of strains that have the same population structure.

To assess the impact of selecting quintessents strains on the extent of recombination, the Phi statistic was computed from multiple sequence alignments for each of the 898 core genes in the data sets: all nt, all aa and quint aa. The results are in Table 1. Each cell in Table 1 shows the number of genes/proteins (out of 898) that fail to reject the null hypothesis of nonrecombination (at the given Phi threshold). That is, they are presumed to be nonrecombinant.

**Table 1:** Counts of genes/proteins whose Phi *p*‐value is greater than designated threshold, thus failing to reject null hypothesis of recombination

Strains set	Count of genes (*N* = 898)			
	Phi > _0.1	Phi > _0.5	Phi > _0.01	≤1 Site (0 sites)
quint aa	482	562	693	10 (4)
all aa	439	518	633	4 (0)
all nt	150	187	255	0 (0)

It is clear from the data in Table 1 that simply moving from nucleotide to amino acid concatenations can significantly reduce the level of apparent recombination. For example, in the all nt data set, at the 0.1 Phi threshold, only 150 genes were found not to be recombinant (i.e., failing to reject the null hypothesis), compared with 439 of the corresponding proteins from the all aa data set. This is most likely due to the buffering provided by the redundancy in the amino acid codon translation table versus the input nucleotide sequences, particularly at the highly variable third codon positions.

Turning to the comparison of amino acid sequences from quintessent strains versus corresponding sequences from nonquintessent strains, use of quintessents further increased the number of nonrecombinant sequences to 482, which is a statistically significant increase (*p* = .0018, on a binomial distribution statistic). This suggests that much of the recombination evident at the nucleotide sequence level may be relatively recent recombination noise, and the move to amino acid sequences—and, particularly, amino acid sequences from quintessent strains—brings us closer to ancient recombinations that have been preserved in the population due to founder effects. However, even after the selection of quintessent strains, certain genes/proteins will exhibit some recombination signal (at thresholds described above), so these have been omitted from the quintessent concatenations.

In view of these results, it is worth turning our attention to the corresponding MLST concatenations, which have been the foundation of many previous analyses; the Phi *p*‐value was 0, comprehensively rejecting the null hypothesis of nonrecombination. In other words, the MLST gene fragment concatenations are overwhelmingly recombinant.

### 3.4 Using quintessents to build phylogenetic trees

The quintessent strains and their STRUCTURE groups are denoted using ‘*’ in Tables S1 and S2. Of the 185 strains in the starting set, 92 strains were removed as they are likely to be hybrid strains resulting from local recombinations between *H. pylori* strains, for which there was limited evidence of preservation in a significant sub‐population. Thus, we lost a significant proportion of the hpEurope strains, where only 16 out of 89 strains were selected. We assume that this is because of the considerable history of human migration across Europe (Lazaridis et al., 2014). Similarly, both of the hpAsia2 strains sequenced for this study could not form a separate group with probability 0.75 in 60% of the runs and therefore were removed. Given that hpAsia2 has been identified as a sub‐population in other studies, the disappearance of these two representatives may simply be due to both of these examples being recombinant. Similarly, the reference strains used in this study, 26695, G27, Pecan4, J99 and India7, also failed to be included as quintessents, suggesting they do not fully represent the sub‐populations they are generally associated with, for example, hpEurope, in the case of 26695.

**Figure 4:**
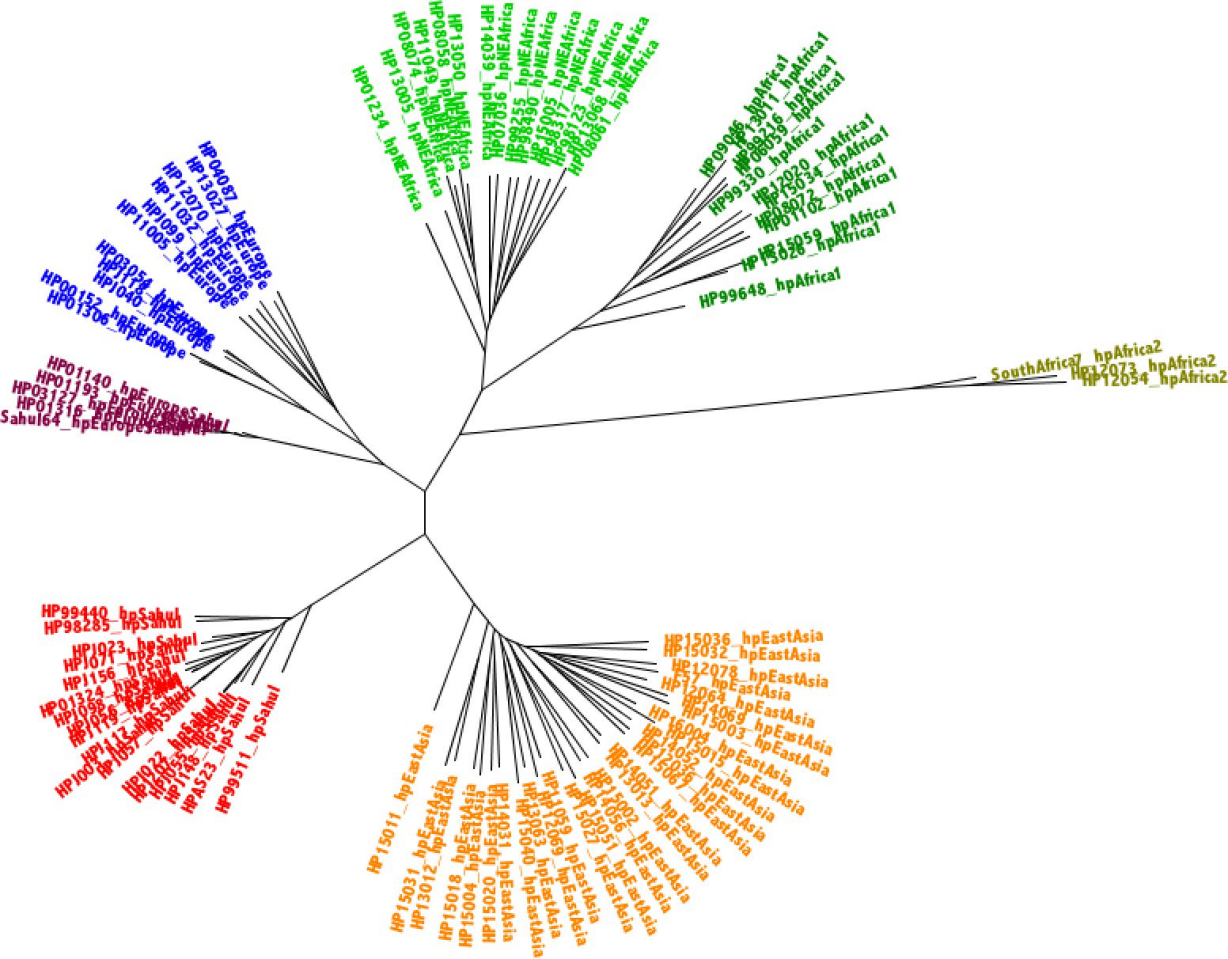
93 Quintessent strain tree. A phylogenetic tree was created by ExaML, based on concatenations of 472 minimally recombinant proteins from 93 strains that were found to be quintessents. A starting tree was created using Mega. Five hundred bootstrapped trees were computed, with the best (i.e., lowest absolute value log likelihood) shown here. The taxa have been labelled, both in the labels and by colour, according to the highest percentage STRUCTURE group, based on the best (lowest absolute value log likelihood) of 12 runs for k = 7 bins (based on suggestion from Moodley et al. (2009)). The clade colours are as follows: hpAfrica2 (olive), hpNEAfrica (bright green), hpAfrica1 (dark green), hpEurope (blue), hpEastAsia (orange) and hpSahul (red). The sub‐clade of hpSahul that we now discover is a new clade, hpEuropeSahul, is shown in maroon

A phylogenetic tree created using the 472 minimally recombinant proteins from these 93 strains (Figure 4) showed a clustering pattern that is similar to trees obtained from the core genomes and core proteomes, but much more clearly delineated. The five quintessent strains isolated from the Aboriginal Australian individuals—originally characterized as hpSahul based on MLST data, but now assigned to a separate clade closer to hpEurope based on core genome and quintessent phylogenies—together suggest a speciation event where hpEurope strains have recombined with hpSahul strains to form a new sub‐population. Therefore, based on the STRUCTURE and phylogenetic data, we have named this new clade hpEuropeSahul.

hpEuropeSahul strains are distinct from both of the parent clades, as their core genes are a mosaic of a majority of genes which are closer in phylogenetic distance to hpEurope and a smaller number of genes which are closer to hpSahul. Specifically, of the 898 core genes, for 662 genes hpEuropeSahul clade is closer to the nearest hpEurope than the nearest hpSahul. For 125 genes, the nearest hpSahul is closer, and for 111 genes, the hpEuropeSahul clade appears equidistant to the parent clades. Given that the strains were obtained from unrelated individuals, we can assume that this clade is being stably inherited. In terms of the evolutionary biology of hpEuropeSahul compared with hpSahul, it is clear that the speciation event that gave rise to hpSahul happened 65 kya, when Aboriginal Australians first migrated to the continent of Australia (then Sahul). What is less clear is when hpEuropeSahul arose, but we assume it is of recent origin, reflecting the colonization of Australia by European settlers around 200 years ago. There is some support for this in the locations where the quintessent strains from Aboriginal Australians were collected. Thirteen quintessent hpSahul strains were collected from the remote settlement of Jigalong (Windsor et al., 2005), one from Alice Springs and four from Perth. By contrast, all five hpEuropeSahul strains were collected from Perth.

### 3.5 Comparing the quintessents method to ClonalFrameML

Table 2 summarizes the data related to cladistic information for the original nucleotide data set (all nt), which contains a considerable level of recombination, together with data from the same data set once it had been processed by ClonalFrameML (all nt cf) and 93 sequences drawn from all nt, corresponding to the strains whose core genomes were identified as being quintessents (quint nt). Unlike its parent data set, the all nt cf data set has a Phi *p*‐value of 1.0, so is clearly nonrecombinant, but the sequences are now much shorter than the parent sequences (or those in quint nt), and the number of informative sites was decreased from 272,018 in the parent set to 224,461 in quint nt to 188,404 in all nt cf. The total entropy score (in bits) in fact increases slightly in quint nt over the parent, presumably because the splits between strains induced by the different sites are more balanced (i.e., half the strains have one nucleotide, while the other have a different nucleotide) in the quintessent data set, which reflects the smaller number of larger clades. On the other hand, when duplicate sites with the same split are removed, there is a significant drop in the dCITE score (Wise, 2016). This is not seen in the all nt cf set, which ends up having the same score as the parent. What the drop reflects is linkage disequilibrium, which we know exists in *H. pylori*, so the fact that the drop is far less in the all nt cf set suggests that that signal is being disrupted by the nonquintessent strains. It should also be noted that invariant sites have been removed from the all nt cf set, which precludes the use of substitution models involving a percentage of invariant sites. It also precludes use of codon‐based models that are generally superior to single nucleotide substitution models (Shapiro, Rambaut, & Drummond, 2006).

**Table 2:** Informative sites and cladistic information content (measured by dCITE scores) for all nt data set, all nt processed by ClonalFrameML and quintessent nucleotide data set drawn from aa nt

	Metric nt	Strains set	
		all nt	all nt cf quint
Length (nt)	828,027	188,404	828,027
Informative sites	272,018	188,404	224,461
Total entropy (bits)	89,870	84,489	90,635
dCITE (bits)	82,526	82,526	75,312

TreeCmp was used to compare the trees produced by the ClonalFrameML‐derived data set (all nt cf), the quintessent nucleotide tree (quint nt) and the amino acid quintessent data set involving minimally recombinant genes (quint aa). The results for four different tree‐difference metrics are shown in Table 3, with visualizations of the trees available as Figures S1 and S2. The TreeCmp prune option was used to enable comparisons of trees with different counts of taxa—the quintessent sets have 93 taxa versus the other data sets' 185—so only the shared taxa are compared. Each of these trees was compared to all the others described above. The first thing that emerges from Table 3 is that, based on 93 common strains, the trees from the all nt cf and quint aa data sets are reasonably similar. However, viewed from the tree due to the quint aa data set, the quint nt data set is closer, despite having been based on concatenations of 898 nucleotide sequences rather than 472 amino acid sequences. The similarity is quite evident in the visualizations of the respective trees.

**Table 3:** All‐against‐all comparison, using TreeCmp, of trees produced all 6 data sets all nt, all aa, mlst and quint aa, all nt cf and quint nt, with the focus on the trees from the ClonalFrameML‐derived data set (all nt cf)

Set1	Set2	Common taxa	Tree‐difference metrics			
			R‐F	MatchingSplit	PathDifference	Quartet
all nt cf	all nt cf	185	0	0	0	0
all nt cf	quint nt	93	24	100	103.9711	122,122
all nt cf	all nt	185	12	109	131.5979	210,794
all nt cf	quint aa	93	37	156	138.9676	223,553
all nt cf	all aa	185	88	436	386.3832	2,258,933
all nt cf	mlst	185	147	1,121	820.1683	12,265,590
quint nt	quint nt	93	0	0	0	0
quint nt	all nt cf	93	24	100	103.9711	122,122
quint nt	quint aa	93	34	118	136.8722	125,298
quint nt	all nt	93	25	105	106.7895	129,569
quint nt	all aa	93	37	206	162.8435	246,198
quint nt	mlst	93	67	372	281.0125	617,813
quint aa	quint aa	93	0	0	0	0
quint aa	quint nt	93	34	118	136.8722	125,298
quint aa	all nt cf	93	37	156	138.9676	223,553
quint aa	all nt	93	38	151	131.1106	223,594
quint aa	all aa	93	41	211	160.1187	251,317
quint aa	mlst	93	67	364	280.7526	604,579

Note

The quintessent data sets involve 93 strains, while the other data sets involve 185 strains. All the metrics are difference metrics, so a distance of 0 implies identical sequences.

## 4 CONCLUSIONS

Our study has described a new method for taxon selection based on taking the strains whose genomes have minimal evidence of recombination. These exemplar strains, which we have called quintessents, represent particular sub‐populations of the input strains. In addition, by moving from nucleotide to amino acid data, and through use of quintessents, we have shown that recombination noise can be greatly reduced, exposing more clearly ancient recombination events that are evident as speciation events. As a demonstration of the new approach, from a starting set of 177 new *H. pylori* genomes plus 8 from the literature, we found 93 quintessents representing 7 *H. pylori* sub‐populations, including a new Sahul sub‐population that has arisen as a result of a recombination event involving hpEurope and hpSahul strains, that is now being stably inherited. Finally, this study has provided further evidence that, in order to get better resolution in phylogenetic analyses, one needs to include more genes than the conventional MLST concatenations, and those genes need to be minimally recombinant. For future work, a more rigorous method, perhaps using an information‐theoretic metric, is required for selecting the quintessent bin probability and percentage of run values.

## CONFLICT OF INTEREST

Barry J. Marshall is medical director of Tri‐Med (http://www.trimed.com.au), a Perth company which distributes diagnostic tests for *Helicobacter pylori* (‘PYtest’ urea breath tests and ‘CLOtest’ biopsy rapid urease test) and marketing orphan drugs (bismuth subcitrate, tetracycline, furazolidone and rifaximin).

## Data Availability

The genomes are available as NCBI Bioproject PRJNA374603. A spreadsheet with the STRUCTURE runs for K = 11 can be downloaded from the University of Western Australia Repository, https://doi.org/10.26182/5d64e7694a120 (Wise, 2019).
