# Supplementary material for: A novel taxon selection method, aimed at minimizing recombination, clarifies the discovery of a new sub‐population of Helicobacter pylori from Australia
Source: Evol Appl. 2019 Sep 18;13(2):278–89. doi: 10.1111/eva.12864 (PMC6976958; doi:10.1111/eva.12864)

Supplementary Data

Table S1. List of genomes sequenced in this project. The data includes the MLST type inferred from the highest scoring bin of the best K=7 bins STRUCTURE run, the number of contigs, the size of the genome, the number of coding sequences, the times coverage of the genome and the G+C content. (K=7 is due to Moodley et al, (2009).) Quintessent strain identifiers are preceded with a *. The hpSahul strains shown in red were found to be hpSahul by MLST, but are now part of the new hpEuropeSahul clade.

SampleID	MLST	Contigs	Bases	CDS	Coverage	G+C%
*HP00152	hpEurope	78	1697338	1590	113	38%
HP00192	hpEurope	49	1652574	1514	82	38%
HP00248	hpNEAfrica	36	1711539	1563	103	38%
HP00260	hpNEAfrica	37	1623837	1494	96	38%
*HP01102	hpAfrica1	33	1661360	1513	89	39%
*HP01140	hpSahul	61	1689516	1556	101	38%
*HP01193	hpSahul	39	1644010	1520	220	38%
*HP01234	hpNEAfrica	45	1686541	1545	128	38%
*HP01306	hpEurope	48	1666338	1548	132	38%
*HP01316	hpSahul	45	1685475	1549	69	38%
*HP01324	hpSahul	27	1539500	1411	258	38%
HP01330	hpEurope	44	1713886	1580	70	38%
HP02140	hpEurope	58	1661330	1529	91	38%
*HP03054	hpEurope	44	1677093	1523	79	38%
*HP03127	hpSahul	33	1675165	1543	209	38%
HP03218	hpNEAfrica	34	1671401	1530	484	38%
HP04041	hpNEAfrica	39	1702667	1555	101	38%
HP04042	hpEurope	27	1627702	1500	116	38%
HP04057	hpEurope	34	1608521	1457	485	38%
HP04086	hpEurope	24	1650009	1537	91	38%
*HP04087	hpEurope	39	1653069	1514	44	38%
HP05044	hpNEAfrica	43	1659886	1518	514	38%
HP06038	hpEurope	36	1666103	1545	276	38%
HP06045	hpAfrica1	29	1673367	1532	388	38%
HP06058	hpEurope	46	1674509	1549	208	38%
*HP06059	hpAfrica1	35	1694537	1557	229	38%
HP07019	hpNEAfrica	27	1611406	1479	170	38%
*HP07036	hpNEAfrica	34	1667043	1530	192	38%
HP08031	hpAfrica1	44	1657553	1521	491	38%
*HP08058	hpNEAfrica	28	1573264	1441	239	38%
*HP08061	hpNEAfrica	43	1674312	1541	273	38%
*HP08072	hpAfrica1	30	1657332	1513	491	39%
HP08073	hpNEAfrica	35	1641467	1506	360	38%
*HP08074	hpNEAfrica	29	1595277	1475	247	38%
*HP09046	hpAfrica1	47	1653875	1514	159	38%
HP11004	hpEurope	32	1602182	1490	118	38%
*HP11005	hpEurope	34	1601647	1490	114	38%
HP11011	hpNEAfrica	42	1678449	1561	82	38%
HP11013	hpNEAfrica	35	1646004	1521	111	38%
HP11020	hpNEAfrica	44	1642462	1536	145	38%

*HP11032	hpEurope	36	1650574	1552	97	38%
HP11037	hpNEAfrica	31	1680812	1546	76	38%
HP11042	hpNEAfrica	29	1670875	1550	185	38%
HP11043	hpEurope	38	1660492	1526	129	38%
*HP11049	hpNEAfrica	50	1578457	1447	103	38%
HP11054	hpEurope	30	1611136	1479	116	38%
HP11055	hpEastAsia	29	1613753	1481	659	38%
*HP11059	hpEastAsia	38	1666250	1549	70	38%
HP12002	hpNEAfrica	21	1606804	1499	214	38%
HP12014	hpEurope	31	1590893	1483	96	38%
HP12019	hpEurope	34	1645725	1522	116	38%
*HP12020	hpAfrica1	33	1625324	1499	172	39%
*HP12036	hpEastAsia	32	1630523	1529	98	38%
HP12038	hpNEAfrica	32	1682424	1550	168	38%
HP12053	hpNEAfrica	32	1642664	1520	102	38%
*HP12054	hpAfrica2	32	1606804	1473	89	38%
HP12059	hpEurope	35	1657768	1519	111	38%
HP12060	hpEurope	38	1606880	1466	91	38%
*HP12064	hpEastAsia	26	1601932	1494	89	38%
HP12068	hpNEAfrica	31	1636516	1515	157	38%
*HP12069	hpEastAsia	36	1622260	1480	73	38%
*HP12070	hpEurope	38	1577110	1443	32	39%
*HP12073	hpAfrica2	36	1633208	1495	196	38%
HP12077	hpNEAfrica	27	1625750	1494	144	38%
*HP12078	hpEastAsia	34	1646065	1545	78	38%
*HP13005	hpNEAfrica	38	1606375	1480	93	38%
HP13007	hpEurope	36	1633616	1493	123	38%
HP13009	hpNEAfrica	46	1681785	1547	79	38%
*HP13011	hpAfrica1	44	1624449	1489	126	38%
*HP13012	hpEastAsia	28	1660361	1551	134	38%
*HP13013	hpEastAsia	38	1688714	1577	79	38%
HP13021	hpNEAfrica	34	1604304	1501	153	38%
HP13022	hpEurope	45	1621499	1493	105	38%
HP13024	hpEurope	46	1668789	1539	146	38%
HP13025	hpEurope	26	1654769	1527	145	38%
*HP13026	hpAfrica1	50	1704151	1553	176	38%
*HP13027	hpEurope	59	1612025	1504	92	38%
HP13028	hpEurope	38	1677419	1568	146	38%
HP13029	hpEurope	30	1590787	1466	141	38%
HP13031	hpEastAsia	47	1614574	1490	69	38%
HP13033	hpEurope	46	1676018	1539	153	38%
*HP13050	hpNEAfrica	31	1606945	1484	106	38%
HP13054	hpEurope	29	1604578	1481	157	38%
HP13056	hpEurope	37	1615355	1485	103	38%
HP13061	hpEastAsia	51	1734529	1620	102	38%
*HP13063	hpEastAsia	24	1643557	1542	99	38%
HP13064	hpNEAfrica	46	1692568	1545	74	38%

*HP13068	hpNEAfrica	24	1666480	1530	181	38%
HP13072	hpAfrica1	39	1702450	1556	167	38%
HP14021	hpNEAfrica	34	1652413	1510	153	38%
HP14023	hpAfrica1	29	1677416	1542	196	38%
*HP14031	hpEastAsia	24	1591184	1485	158	38%
HP14036	hpEurope	46	1654846	1525	196	38%
*HP14039	hpNEAfrica	38	1693832	1564	157	38%
HP14048	hpNEAfrica	42	1629216	1470	149	38%
HP14050	hpEurope	32	1609742	1464	121	38%
*HP14051	hpEastAsia	24	1581578	1484	171	38%
*HP14052	hpEastAsia	32	1577640	1487	78	38%
HP14054	hpNEAfrica	28	1607436	1490	138	38%
*HP14056	hpEastAsia	28	1642674	1524	158	38%
HP14065	hpEurope	34	1659861	1519	172	38%
*HP14069	hpEastAsia	39	1646965	1537	227	38%
*HP15002	hpEastAsia	27	1609232	1511	123	38%
*HP15003	hpEastAsia	31	1624824	1516	76	38%
*HP15004	hpEastAsia	24	1678902	1556	151	38%
*HP15005	hpNEAfrica	44	1584917	1450	92	38%
HP15006	hpEurope	36	1679866	1554	176	38%
*HP15011	hpEastAsia	31	1679676	1563	176	38%
HP15012	hpEurope	40	1626944	1509	204	38%
HP15013	hpNEAfrica	47	1590565	1495	252	38%
*HP15015	hpEastAsia	37	1571234	1483	191	38%
*HP15018	hpEastAsia	21	1570603	1472	137	38%
*HP15020	hpEastAsia	27	1599714	1493	80	38%
HP15022	hpEurope	53	1685711	1551	75	38%
HP15025	hpNEAfrica	38	1712317	1563	192	38%
HP15026	hpEurope	43	1721368	1591	139	38%
*HP15027	hpEastAsia	26	1609042	1494	140	38%
HP15028	hpEurope	42	1658379	1538	224	38%
*HP15031	hpEastAsia	38	1632267	1501	113	38%
*HP15032	hpEastAsia	29	1581242	1467	98	38%
HP15033	hpEurope	54	1597292	1480	111	38%
*HP15034	hpAfrica1	45	1656103	1512	141	39%
HP15035	hpNEAfrica	60	1631909	1504	67	38%
*HP15036	hpEastAsia	43	1562337	1453	95	38%
HP15039	hpEurope	86	1615038	1482	93	39%
*HP15040	hpEastAsia	19	1623997	1508	164	38%
HP15044	hpEurope	46	1668737	1538	199	38%
HP15050	hpEurope	42	1684919	1550	264	38%
*HP15051	hpEastAsia	26	1637055	1530	264	38%
HP15054	hpAsia2	34	1637741	1508	317	38%
HP15058	hpEurope	28	1574984	1460	201	38%
*HP15059	hpAfrica1	57	1688297	1524	45	38%
HP15060	hpEurope	36	1647766	1513	177	38%
*HP15067	hpEastAsia	25	1607445	1499	663	38%

HP16001	hpEurope	43	1729834	1594	654	38%
*HP16004	hpEastAsia	21	1546654	1460	618	38%
HP16008	hpNEAfrica	32	1676033	1548	592	38%
HP97011	hpNEAfrica	23	1577942	1479	160	38%
*HP98123	hpNEAfrica	42	1661589	1530	109	38%
*HP98285	hpSahul	31	1538879	1413	163	38%
*HP98317	hpNEAfrica	36	1629300	1495	131	38%
*HP98490	hpNEAfrica	38	1715827	1591	79	38%
*HP99216	hpAfrica1	46	1655363	1522	150	38%
HP99244	hpAsia2	31	1639801	1524	136	38%
*HP99255	hpNEAfrica	28	1590821	1446	156	38%
HP99316	hpEurope	46	1688494	1561	88	38%
*HP99330	hpAfrica1	38	1622875	1493	114	39%
HP99392	hpNEAfrica	31	1658415	1542	109	38%
*HP99440	hpSahul	26	1526286	1404	218	38%
*HP99511	hpSahul	47	1555060	1430	36	38%
HP99521	hpAfrica1	43	1651300	1521	104	38%
HP99647	hpEurope	30	1627665	1475	100	38%
*HP99648	hpAfrica1	29	1649856	1517	105	38%
HP99689	hpNEAfrica	47	1642154	1508	112	38%
HPAS14	hpEurope	36	1604234	1479	54	38%
*HPAS23	hpSahul	51	1545003	1427	108	38%
*HPJ003	hpSahul	28	1543937	1405	173	38%
HPJ013	hpEurope	51	1690070	1547	99	38%
*HPJ022	hpSahul	41	1549623	1425	139	38%
*HPJ023	hpSahul	47	1550130	1410	156	38%
HPJ024	hpEurope	47	1712168	1594	65	38%
HPJ025	hpEurope	53	1710240	1582	59	38%
*HPJ040	hpEurope	47	1674590	1529	90	38%
HPJ050	hpEurope	68	1710854	1581	100	38%
*HPJ055	hpSahul	33	1554795	1430	165	38%
*HPJ056	hpSahul	52	1557485	1423	105	38%
*HPJ057	hpSahul	61	1549013	1413	134	38%
*HPJ071	hpSahul	32	1549498	1428	79	38%
*HPJ098	hpSahul	20	1529676	1404	88	38%
*HPJ099	hpEurope	28	1607159	1496	110	38%
*HPJ117	hpSahul	28	1542229	1414	130	38%
*HPJ118	hpEurope	42	1674504	1529	108	38%
*HPJ119	hpSahul	61	1563128	1427	94	38%
*HPJ148	hpSahul	31	1554757	1438	52	38%
*HPJ156	hpSahul	40	1592098	1458	72	38%
HPJ165	hpEurope	55	1722574	1580	93	38%
*HPJ207	hpSahul	51	1553223	1430	107	38%

Table S2. List of complete genomes from the literature, obtained from NCBI. The data includes the MLST type inferred from the highest scoring bin of the best K=7 bins STRUCTURE run, the size of the genome, the number of coding sequences, the times coverage of the genome and the G+C content. (K=7 is due to Moodley et al, (2009).) Quintessent strain identifiers are preceded with a *. The hpSahul strain shown in red was found to be hpSahul by MLST, but is now part of the new hpEuropeSahul clade.

SampleID	MLST	Bases	CDS	G+C%
26695	hpNEAfrica	1667867	1577	39%
*F57	hpEastAsia	1609006	1527	39%
G26	hpNEAfrica	1652982	1566	39%
India7	hpAsia2	1675918	1568	39%
J99	hpAfrica1	1643831	1504	39%
PeCan4	hpEurope	1629557	1521	39%
*Sahul64	hpSahul	1665382	1563	39%
*SouthAfrica7	hpAfrica2	1653913	1542	38%

Figure S1. Tree created by ExaML from the quint_nt data set (sequences corresponding to the quintessent strains taken from the parent nucleotide data set all_nt. The tree has been visualised using FigTree.

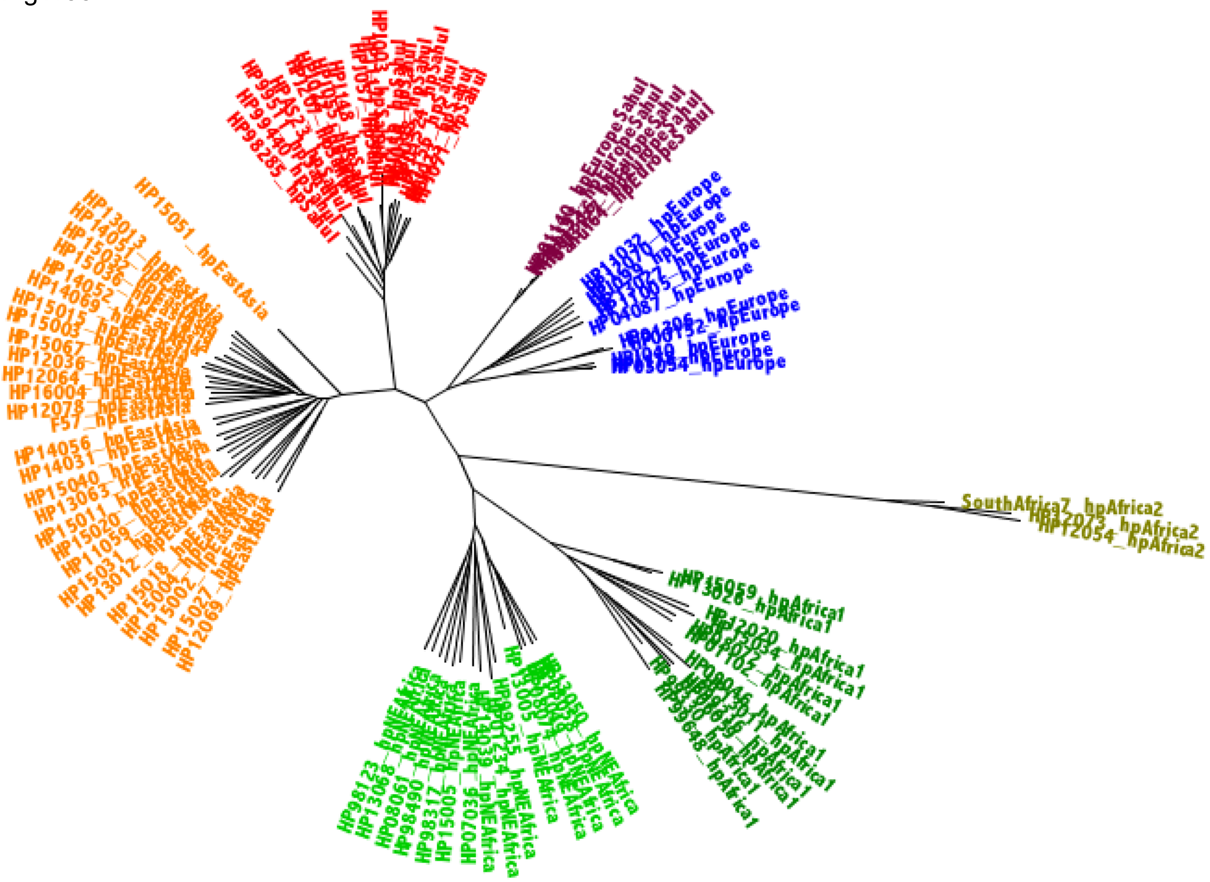

Figure S2. Tree created by ExaML from the all_nt_cf data set ClonalFrameML applied to the parent nucleotide data set all_nt. The tree has been visualised using FigTree.

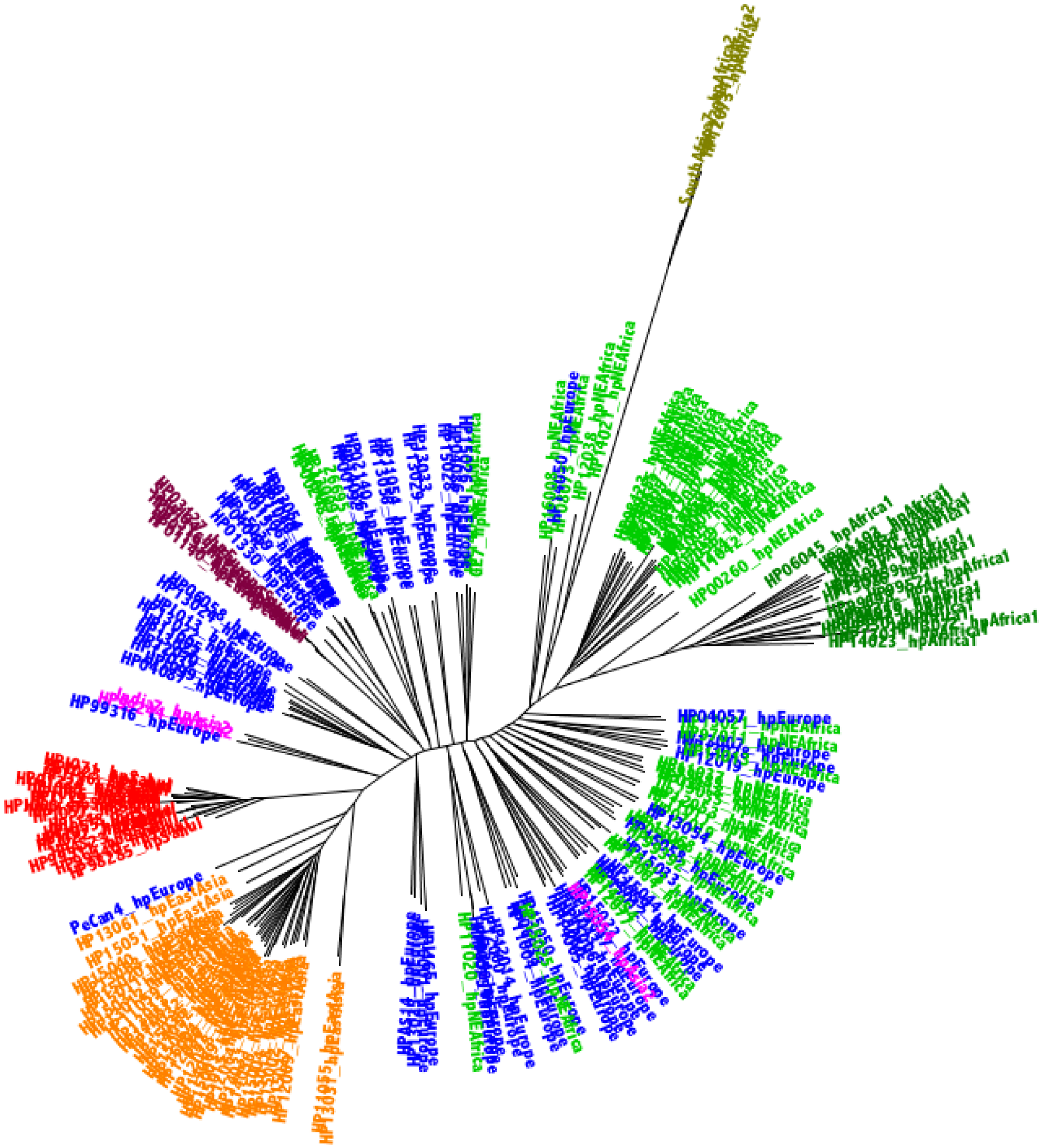

Supplement: Supplementary file 1 [file EVA-13-278-s001.pdf]
